# Comparative Evaluation of Benchtop and Portable Near-Infrared Spectrometers for Predicting the Age and Blood Feeding History of *Aedes aegypti*

**DOI:** 10.3390/insects16111143

**Published:** 2025-11-07

**Authors:** Ayako Takahashi, Elvis Aquino Flores, Rafael Maciel-de-Freitas, Tharanga Kariyawasam, Maggy T. Sikulu-Lord

**Affiliations:** 1 School of the Environment, The University of Queensland, Brisbane, QLD 4072, Australiae.aquinoflores@student.uq.edu.au (E.A.F.);; 2 Laboratório de Mosquitos Transmissores de Hematozoários, Instituto Oswaldo Cruz, Fiocruz, Rio de Janeiro 21040-360, Brazil; 3 Department of Entomology and Arbovirology, Bernhard Nocht Institute for Tropical Medicine, 20359 Hamburg, Germany; 4 NIRSID Consortium, Brisbane, QLD 4072, Australia

**Keywords:** mosquitoes, near-infrared spectroscopy, age grading, blood feeding history, *Aedes aegypti*

## Abstract

**Simple Summary:**

Vector control programs need to conduct routine surveillance to determine the efficacy of control interventions. The near-infrared (NIR) spectroscopy technique combined with machine learning algorithms has been demonstrated as a rapid technique for characterizing mosquito samples into age, species and infection status. However, more affordable spectrometers that have recently been developed are yet to be investigated for this purpose. This study is a comparative assessment of a benchtop spectrometer traditionally used to characterize mosquitoes with wavelength ranging from 350 to 2500 nm (Labspec 4i), with a handheld spectrometer (NIRvascan) with wavelength ranging from 900 to 1700 nm for age grading and for determining the blood feeding history of laboratory-reared female *Aedes aegypti* mosquitoes. Our findings indicate that handheld spectrometers can predict the age of mosquitoes and classify them into two groups (< or ≥10 days) with a similar predictive accuracy as the traditionally used benchtop spectrometers. If further developed, portable NIR instruments could potentially be used to characterize mosquito populations in the field. The next step is to conduct a field study to determine the capacity of these portable spectrometers to analyze wild mosquito samples, preferably onsite.

**Abstract:**

This study is a comparative assessment of a more affordable handheld spectrometer (NIRvascan) with the traditional Labspec 4i spectrometer for predicting the chronological age and blood feeding history of female *Aedes aegypti* mosquitoes reared in the lab. Three separate cohorts of laboratory-reared *Ae. aegypti* mosquitoes were reared and collected at three age groups (1-, 10- and 17-days old). A model developed using Artificial Neural Networks (ANN) with spectra collected by the Labspec 4i NIR spectrometer predicted the age of *Ae. aegypti*, classifying them into two groups (< or ≥ 10 days) with a predictive accuracy of 94% (N = 366) whereas an ANN model developed using spectra collected by the NIRvascan spectrometer predicted the age of *Ae. aegypti* mosquitoes, classifying them into the same age group with a predictive accuracy of 90% (N = 290). ANN models developed for predicting the blood feeding history of mosquitoes were 82.8% (N = 308) and 71.4% accurate (N = 300) when Labspec 4i and NIRvascan were used, respectively. This is the first study to demonstrate that a handheld NIR instrument operated by a smart phone could potentially be used for predicting entomological parameters of mosquitoes.

## 1. Introduction

*Aedes aegypti* is the primary vector for multiple arboviruses transmitted to humans including dengue virus (DENV), yellow fever virus (YFV), Ross River virus (RRV), chikungunya virus (CHIKV), and Zika virus (ZIKV). With an estimated 300 million annual cases, DENV is the most prevalent and widespread arbovirus placing half of the world’s population at risk [1,2,3]. In 2019, reported incidents of DENV spread across 129 nations [3]. Moreover, DENV is rapidly spreading to areas where it was previously eliminated. This is due to an exponential population growth rate, changes in climate conditions and urbanization which makes new areas suitable habitats for mosquito breeding [4,5]. Vector control strategies to minimize host–vector contact is the most effective way to control the transmission of vector-borne diseases.

Determining the age of mosquitoes facilitates our understanding of their capacity to transmit pathogens [6]. This is due to the lengthy period required by pathogens to develop inside mosquitoes. For example, mosquitoes must acquire pathogens from an infected person, support the pathogen’s incubation period, and then find a susceptible host to transmit the pathogen. At 30 °C and 32 °C, DENV-2 takes 12 and 7 days, respectively, to fully develop within mosquitoes [7], while CHIKV takes approximately 2–7 days [8]. Determining whether a mosquito has previously had a blood meal and how many gonotrophic cycles it has undergone is also a good indicator of vector competence [9]. This is because a mosquito can only transmit a pathogen on its second or subsequent feed. However, estimating the gonotrophic history of mosquitoes is a difficult undertaking. Traditionally, ovary dissections are used to assess the gonotrophic histories of mosquitoes with the Detinova technique being used to identify whether a mosquito had previously laid eggs or not [10]. The more advanced Polovodova’s technique is used to estimate the number of gonotrophic cycles of a mosquito [11,12]. While cost-effective, ovary dissections require skilled personnel whereas dissections are technically demanding and time consuming for large sample sizes. These issues are compounded by the presence of a varying number of dilatations per ovariole in females believed to have similar physiological age [13]. This includes the observation of additional dilatations than the number of gonotrophic cycles a mosquito has undergone [14]. Over the last decade, a rapid and reagent free technique that uses the near-infrared (NIR) region of the electromagnetic spectrum has been demonstrated to be a viable alternative to ovary dissections.

Near-infrared spectroscopy (NIRS) technique involves shining a beam of non-destructive near-infrared light on mosquitoes where some of the light is absorbed and some is reflected. The reflected light is characteristic of chemical and physiological changes happening inside the mosquito and can be used to characterize a mosquito into species type, age and infection status. NIRS is rapid, non-destructive, requires minimal sample preparation, and eliminates the need for reagents, allowing the large-scale analysis of mosquito samples over a short period of time. Only one spectral signature is collected, and that signature can be used to predict multiple parameters such as age, species identity or infection status following the development of predictive models. Several studies have demonstrated that NIRS can predict the age of major African malaria vectors, *Anopheles gambiae* and *An. arabiensis* [15,16,17] as well as the age of *Ae. aegypti* [18] and *Ae. albopictus* [19], the primary and secondary vectors of arboviruses, respectively, in lab settings. Studies under semi-field [16,20] and field conditions [16,21,22,23] have also been conducted to predict the age and species of *An. gambiae*, *An arabiensis* and *Ae. aegypti*. NIRS has also been used to detect DENV [24], ZIKV [25,26], CHIKV [25] and *Wolbachia* [25,27] in *Ae. aegypti* mosquitoes, *Plasmodium* in *An. gambiae* mosquitoes [28] and *Trypanosoma cruzi* in Triatomine bug species [29].

However, previous studies have primarily used the Labspec 4i benchtop spectrometer (Malvern Panalytical, Malvern, UK), which operates across a wavelength of 350 to 2500 nm. Although the Labspec 4i is accurate for predicting the age, species and infection of mosquitoes, the overlay cost for a spectrometer (approximately USD60000) limits its uptake, particularly in resource-limited settings. While NIRS has proven effective using benchtop spectrometers, more affordable and portable spectrometers are yet to be assessed for entomological characterization.

This study compared the accuracy of the Labspec 4i spectrometer with the accuracy of a portable, and relatively affordable spectrometer known as NIRvascan with an operating wavelength of 900–1700 nm (Allied Scientific Pro, Gatineau, QC, Canada) for predicting the age and parity of *Ae. aegypti* mosquitoes in the laboratory. We also determined the cost, scanning time, and operational logistics of both spectrometers.

## 2. Materials and Methods

### 2.1. Mosquito Rearing, Feeding and Sampling

Female *Ae. aegypti* mosquitoes were reared at standard insectary conditions, i.e., at 27 °C, relative humidity of 70–80% and a photoperiod of a 12:12 light cycle. Three different cohorts with approximately 2500 *Ae. aegypti* eggs each, flooded in trays of water were reared at three different time points. Approximately 500 larvae were reared per tray and fed with fish food (Kyorin Food industries, Kyorin, Hikari, Japan). Approximately 800 pupae were transferred into four cages for emergence. Two of the cages were set aside for sugar feeding and two were used exclusively for blood feeding experiments. All adult mosquitoes were provided with access to 10% sugar solution ad libitum. Prior to blood feeding, female mosquitoes at 5- and 12-days old were starved for 24 h and blood-fed with human blood following the human ethics protocol approved by the University of Queensland (Approval number 2020001077). Three different treatment groups of mosquitoes were collected for scanning as follows; control (unfed) (1-, 10-, and 17-days old); single blood-fed mosquitoes (10- and 17-days old) collected after oviposition; and twice blood-fed mosquitoes (17-days old) collected after oviposition. Unfed mosquitoes were separated from fed mosquitoes and added to the cage that was not blood-fed. Following collection, mosquitoes were stored at 4 °C between 5 and 18 days prior to scanning. Table 1 shows a summary of the number of mosquitoes sampled per treatment for the three cohorts.

### 2.2. Mosquito Scanning Using Labspec 4i

**Figure 1:**
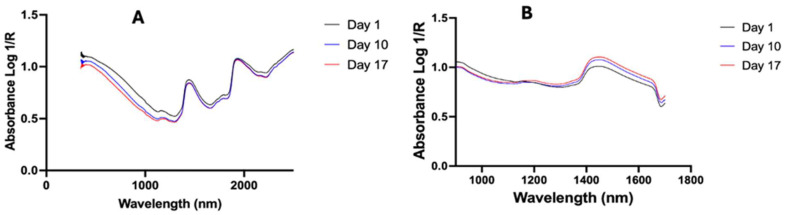
The average raw spectra of 1-, 10-, and 17-day old mosquitoes collected by the Labspec 4i (**A**) with light shining on mosquitoes from above and NIRvascan (**B**) with light shining on mosquitoes from underneath.

To assess the performance of Labspec 4i and NIRvascan spectrometers, each mosquito was scanned first with the Labspec 4i then with the NIRvascan. Scanning with Labspec 4i followed the protocol described by Mayagaya and colleagues [15]. Briefly, the Labspec 4i spectrometer was connected to a computer, and an external fiber optic probe with six illumination fibers was attached (Malvern Panalytical, Malvern, UK). Prior to scanning, the fiber optic probe was positioned 2 mm above the mosquito head and thorax, providing adequate space between the mosquito and the probe. The spectrometer was calibrated (optimized and white-referenced) using a clean spectralon panel and thereafter recalibrated every half an hour during the scanning session. Following calibration, each mosquito was placed on its right side up on the spectralon panel for scanning. Spectral signatures (Figure 1A) were collected by pressing the space bar on the laptop connected to the LabSpec 4i.

### 2.3. Mosquito Scanning Using NIRvascan

Prior to scanning, a NIRvascan spectrometer was connected to a smartphone via Bluetooth using the ISC NIRScan app (InnoSpectra Corporation, Singapore). A whole mosquito was placed on its right side up on the detector, allowing the whole mosquito to be scanned from underneath the infrared light. Unlike Labspec 4i where light is shone on mosquitoes from above, mosquitoes are scanned using NIRvascan with light from below; therefore, the average raw spectra of 1-, 10- and 17-day old mosquitoes scanned by the NIRvascan are inverse to the average raw spectra of similar mosquitoes collected by the Labspec 4i spectrometer due to differences in scanning methods used for the two spectrometers (Figure 1B). Mosquitoes were randomly scanned by both spectrometers by disregarding their age and feeding status.

### 2.4. Data Analysis

JMP 17 Pro (SAS Institute Inc., Cary, NC, USA) was used to train machine learning algorithms for predicting mosquito age and blood-feeding status for both spectrometers. Separate models were developed for spectral data collected using Labspec 4i and the NIRvascan spectrometers. In summary, age prediction models and models for predicting blood feeding history models were developed using mosquitoes from one cohort but representative samples from the remaining two cohorts were added to improve the robustness of the model (Table 2). The two spectrometers cover different spectral ranges (Labspec 4i: 350–2500 nm; NIRvascan: 900–1700 nm) therefore each dataset was analyzed using a region that yielded the highest prediction accuracy for that instrument. This approach reflects the optimal operational condition for each spectrometer, allowing us to evaluate their real-world performance.

For age prediction models, mosquitoes were assigned a value that represented their actual age. Similarly, to predict blood feeding status, a value of “0” was assigned to the spectra of mosquitoes that were unfed (Control), and a value of “1” was assigned to the spectra of mosquitoes that were blood-fed once, and a value of “2” for mosquitoes that blood-fed twice. Artificial neural networks (ANN) and K-fold (K = 5) cross validation were used to analyze raw spectral data with wavelength being treated as an independent factor. Mosquito age and blood feeding status were treated as dependent factors in the model, creating two separate models for predicting the age and blood feeding history for the two datasets. The wavelength that resulted in the most accurate results for age grading and blood feeding history prediction for each spectrometer is shown in Table 2. For predicting both the age and parity status of mosquitoes, the ANN model comprised one hidden layer (TANH = 3), a learning rate of 0.1, and one tour.

To check for overfitting, the resulting ANN model was then applied to a pre-validation set of mosquitoes that were included in the model but were not used in training the model and to a validation set of mosquitoes from other cohorts as shown in Table 2. The accuracy of the models for each spectrometer developed to predict the age, and blood meal history was tested on mosquitoes at 1-, 10-, and 17-days old that were either blood-fed or unfed. ANOVA was used to compare the difference between the age and blood meal predictive accuracies of the two spectrometers.

## 3. Results

### 3.1. Prediction Within the Training Set

Following prediction, mosquitoes were classified into two age (< or ≥10 days old) representing young mosquitoes (1-day old) and old/potentially infectious mosquitoes (≥10-days old) mosquitoes. The most accurate training algorithm for predicting the age of mosquitoes that were scanned with the Labspec 4i and NIRvascan spectrometers was 92.9% and 91.3%, respectively, with 10-day old mosquitoes less accurately predicted by both devices. We used 10 days as a cut off point for age prediction; hence, it was expected that this age group would be the least accurately predicted. The accuracy for predicting very young mosquitoes (1-day old) and old mosquitoes (17-days old) was 100% for both spectrometers.

In terms of blood feeding, the training model using data collected by NIRvascan was generally more accurate (97.4%) than the model developed using Labspec 4i data (94.4%). NIRvascan was 97.8% accurate for predicting older mosquitoes in the 17–18 day age group as having previously received or not received a blood meal, whereas Labspec 4i predicted mosquitoes in the same age group as having received or not received a blood meal with 86% accuracy (Table 3).

### 3.2. Validation Set

#### 3.2.1. Prediction of Age

Generally, age prediction results for the validation set were consistent with the results from the training set. Labspec 4i predicted the age of mosquitoes with an overall accuracy of 94% (N = 366), while the NIRvascan predicted the age of mosquitoes with an overall accuracy of 90% (N = 290). Generally, Labspec 4i performance was not any better in predicting 10–11 day and 17–18 day age groups where accuracies of 87.3% and 97.3%, respectively, were achieved, compared to NIRvascan’s 83.6% and 91.8% for the same age groups. Both spectrometers predicted the average age of 10- and 17-day old mosquitoes as 13 and 14-days old, respectively, and this prediction was not significantly different with two-way ANOVA for mosquitoes that were 10-days old (*p* = 0.5871) or 17-days old (*p* = 0.5586). For mosquitoes within the 1–2 day age group, NIRvascan outperformed Labspec 4i, achieving 100% accuracy compared to Labspec’s 96.3% accuracy. The mean predicted age of 1-day old mosquitoes was 4 days when Labspec 4i was used and 0.7- days old when NIRvascan was used and this prediction was significant between the two groups (*p* < 0.00001). Generally, both spectrometers predicted the age of younger and older mosquitoes more accurately than they did for 10-day old mosquitoes. This is due to the fact that 10 days were selected as a cut off for the correct or incorrect prediction for both spectrometers (Table 4).

#### 3.2.2. Prediction of Blood-Meal History

Models developed were unable to classify mosquitoes into more than one feeding cycle; therefore, a prediction was considered correct if an unfed mosquito was classified as unfed or if a blood-fed mosquito (whether fed once or twice) was classified as blood-fed. Overall, the Labspec 4i predicted the feeding history of mosquitoes with an overall accuracy of 82.8% (n = 308) compared to NIRvascan’s 71.3% (n = 300). Regardless of the age of the mosquitoes, the Labspec 4i spectrometer predicted the feeding status of unfed (0), fed once (1), and fed twice (2) groups into the fed and unfed groups with an accuracy of 77.9%, 88.5%, and 95.8%, respectively, compared to NIRvascan’s 72.4%, 70.5%, and 68.9%, for the same age groups. Regardless of the age of the mosquito analyzed, Labspec 4i was more accurate in predicting whether a mosquito had received a blood meal than when it was unfed. Comparatively, NIRvascan was more accurate in predicting unfed mosquitoes than those previously fed. However, both spectrometers were not able to predict more than one feeding cycle (Table 5).

### 3.3. Effect of Blood Meal on Age Prediction by Both Spectrometers

Using ANOVA, we determined the effect of blood feeding on the age prediction of mosquitoes (Table 6). Mosquitoes that received a blood meal and laid eggs were generally predicted slightly more accurately by both spectrometers than those that did not blood feed. There was a difference between the mean predicted age of mosquitoes that received a blood meal from those that did not receive a blood meal for mosquitoes that were 10-days old (*p* = 0.049) but not for mosquitoes that were 17-days old (*p* = 0.678) for NIRvascan. For the Labspec 4i, blood-fed mosquitoes at 10 days were predicted as 12.3-days old compared to the unfed ones which were predicted as 14.3-days old. This mean predicted age was significantly different (*p* < 0.001). Blood-fed mosquitoes that were 17-days old were predicted as 14.1-days old and this prediction significantly differed from that of unfed mosquitoes at the same age group (*p* = 0.005). This indicates that physiological changes occurring within blood-fed and unfed mosquitoes have an overall effect on the aging process of the mosquitoes, ultimately affecting their age prediction.

### 3.4. Comparative Analysis of Labspec 4i and NIRvascan in Terms of Time, Cost and Operational Complexity

Lastly, we compared the Labspec 4i and NIRvascan spectrometers in terms of outlay costs, spectral range, resolution, and average sampling time. Table 7 provides a summarized overview of these comparisons.

## 4. Discussion

Entomological surveillance is a crucial undertaking in vector control programs as it determines the efficacy of existing interventions. Determining mosquito age structure is one way to assess how well interventions are performing. Traditionally, this is achieved through parity dissections, a time-consuming and unlikely scalable way for large programs. Other techniques such as the assessment of the abundance of transcriptional profiles [30] and changes in cuticular hydrocarbons [31] were developed in the last decade. However, due to their operational complexity and high operational costs, their uptake has been slow. NIRS technique has shown promise for age grading both *Anopheles* and *Aedes* mosquitoes in the lab [15,18], semi-field systems [16] and in the field [21,22,23] using the Labspec 4i. However, affordable spectrometers that have been developed recently are yet to be assessed for this application. This study compared the performance of a portable handheld spectrometer known as NIRvascan and a benchtop spectrometer known as Labspec 4i for analyzing lab reared *Ae. aegypti* mosquitoes to determine their chronological age and blood feeding history. To our knowledge, this is the first study to demonstrate that a handheld NIR instrument shows potential as a viable alternative to the traditional Labspec 4i spectrometer for the rapid characterization of mosquito traits to support entomological surveillance.

The chronological status of mosquitoes was defined using an age threshold of 10 days representing mosquitoes that are less likely to be infectious (<10 days) and more likely to be infectious (≥10 days old), with predictions classified as either younger than 10 days or ≥10 days. In a past study, the Labspec 4i spectrometer accurately differentiated *Ae. aegypti* and *Ae. albopictus* into two age categories with a predictive accuracy of 91% for *Ae. aegypti* [18] and 94% for *Ae. albopictus* [19]. These predictive accuracies are consistent with the accuracy of 94% and 91% achieved by the Labspec 4i and NIRvascan, respectively, under this study. Both spectrometers predicted the age of younger (1-day old) and older (17-days old) mosquitoes more accurately than the middle age group (10-days old) and this was also consistent with the previous studies [18,19]. NIRvascan predicted the age of 1-day old mosquitoes more accurately than the Labspec 4i. One-day old mosquitoes were predicted on average as 0.7 days compared to the prediction of 4 days achieved by the Labspec 4i. The wavelength that was used to predict the age of mosquitoes was within 1050–2350 nm for the Labspec 4i, and 950–1650 nm for the NIRvascan. The high age prediction accuracy achieved by both spectrometers indicates that age-related cuticular changes are predominantly happening within the 900–1650 nm as previously reported in culicoides [32] and that the additional wavelengths offered by the Labspec 4i have very little effect on the overall prediction of chronological age of *Ae. aegypti* mosquitoes. Importantly, NIR comprises repeated overtones and combinational bands which means some of the information within the combination region and 1st overtone (1800–2350 nm) of the Labspec 4i spectrometer is present within the 2nd (1100–1600 nm) or 3rd overtone (900–1400 nm) of the NIRvascan spectrometer. This is good news as it means that more affordable handheld instruments could be used as alternatives to the Labspec 4i for age prediction.

For physiological age prediction, the Labspec 4i spectrometer generally performed better than the NIRvascan with 82% (N = 302) of mosquitoes correctly predicted as either having never blood-fed or as having received at least one blood meal. Additionally, 96% (N = 48) of mosquitoes that received more than one blood meal were correctly predicted in comparison with mosquitoes that fed only once (88.5%, N = 68). Comparatively, NIRvascan differentiated unfed from fed mosquitoes with 71% (N = 300) with the unfed, fed once and fed twice groups predicted with a similar accuracy. The models developed for both spectrometers were unable to predict the number of times a mosquito had received a blood meal. While distinguishing the exact number of blood meals of a mosquito would have been ideal, predicting whether a mosquito had taken at least one blood meal represents a critical step for population-level surveillance and provides a foundation for future studies aimed at discriminating multiple blood meals. The region that was used to predict the blood feeding history of mosquitoes was within 500–2350 nm for the Labspec 4i and 950–1650 nm for the NIRvascan. It is worth noting that analysis on restricted wavelength was not conducted for both spectrometers. It is plausible that hematophagy induces significant structural and biochemical alterations in mosquitoes, manifesting as detectable spectral signatures within the NIR region 1040, 1140, 1200 nm [33] and the visible range (500–750 nm) [34]. The absence of coverage of these wavelengths in the visible region by the NIRvascan system may account for its diminished accuracy in detecting such physiological changes. Nonetheless, if used in the field, the 90% and 71% accuracy achieved by the NIRvascan would be sufficient to estimate changes in population age structure and the degree of exposure of mosquitoes to human and animal hosts due to its capacity to scan a large number of samples.

In addition to assessing their predictive accuracy for these two entomological indicators, we also conducted a comparative analysis of both spectrometers in terms of their portability, cost, resolution and sampling time with the aim of evaluating the feasibility of using NIRvascan as a field-deployable alternative to the Labspec 4i spectrometer. The Labspec 4i spectrometer has a wavelength ranging from visible light region (350 to 700 nm) compared to NIRvascan’s wavelength of 900–1700 nm with a 3 nm resolution in the visible spectrum and 10 nm in the SWIR filters. The signal-to-noise ratio for the Labspec 4i is much higher (1 nm) compared to NIRvascan’s 10 nm resolution. In terms of the sampling speed and depending on the operator’s experience, Labspec 4i was 5 times faster than NIRvascan. It took 5–15 s on average to scan a sample with the Labspec 4i spectrometer and 30 s with the NIRvascan. NIRvascan’s speed is largely due to the logistics involved in positioning samples on the detector and acquiring a spectrum through a mobile phone connected to the spectrometer via Bluetooth. Comparatively, 15–20 samples can be pre-positioned on a spectralon plate of the Labspec 4i directly under an external probe followed by a 5 s spectral acquisition using the space bar on a computer. Despite these limitations, NIRvascan offers attractive advantages, including being highly portable as it only weighs 136 g (roughly the weight of a smartphone), compared to the Labspec 4i, which weighs 5.6 kg (excluding the laptop, 1-m-long probe, a spectralon plate/platform and charging cables). It took 10 min to train students to use NIRvascan and half an hr to use the Labspec 4i. Perhaps the most significant advantage of NIRvascan is its cost: it is approximately 23 times (USD 3000) cheaper than the Labspec 4i (USD 60000).

Our study is subject to several limitations. First, our findings are derived exclusively from a laboratory-reared colony of *Ae. aegypti* with controlled temperature and humidity conditions rather than field-collected populations, which may exhibit varying physiological and behavioral traits. Similarly, experimental conditions were highly controlled which restricts the future application of the tool to the conditions tested in this study and limits applications in natural field environments. Therefore, extrapolating our lab-based observations to field scenarios should be approached with caution, as direct correlations may not hold true due to varying environmental conditions including humidity, temperature larval diets and physiological changes, all of which have been shown to have an effect on the spectral signatures of mosquitoes [22,35]. Future field studies should incorporate these environmental variations into predictive models. Additionally, the comparative analysis between the NIRvascan and Labspec 4i spectrometers was constrained by their non-overlapping spectral ranges, which restricts direct comparisons to shared absorption features. To extend the range of applications of the assessed spectrometers for other mosquito species, models developed under this study could be improved by incorporating the spectra of other mosquito species.

## 5. Conclusions

In summary, both spectrometers predicted the age of lab-reared mosquitoes into young and old age groups, with the handheld spectrometer achieving a similar accuracy to the benchtop instrument at a fraction of the cost. Although a slightly lower predictive accuracy for blood feeding history was achieved by NIRvascan, the device’s portability and affordability suggest strong potential for field application pending further validation with wild-caught mosquitoes. We conclude that the use of NIRvascan for the age prediction of *Ae. aegypti* is consistent with the accuracies of Labspec 4i and if assessed in the field, it could extend the options of NIR spectrometers available for entomological surveillance in diverse settings.

## Figures and Tables

**Table 1:** The number of mosquitoes sampled for each time point, treatment and cohort.

	No. of Mosquitoes Sampled			
Cohort	Unfed	Blood-Fed Once	Blood-Fed Twice	Total
1	127	38	39	204
2	86	20	26	132
3	120	80	40	240
Total	333	138	105	576

**Table 2:** The number of samples used to develop training models for predicting the age and blood feeding of mosquitoes and the wavelength used for developing the models for both parameters.

	Labspec 4i			Region (nm)	NIRvascan		Region (nm)
Age	Cohort	Training	Validation	1050–2350	Training	Validation	950–1650
	1	100	104		14	190	
	2	50	82		82	50	
	3	60	180		190	50	
Blood meal	1	80	124	500–2350	14	190	950–1650
	2	68	64		82	50	
	3	120	120		220 *	60 **	

* Included 30 mosquitoes that were partly blood-fed; ** included 10 mosquitoes that were partly blood-fed.

**Table 3:** Results from the training set indicating the predictive accuracy of the two spectrometers for predicting the age and blood meal history for samples that were used in the training set only.

Age in Days	Predictive Accuracy for Age [N]		Feeding Condition	Predictive Accuracy for Blood Feeding [N]	
	Labspec 4i [N]	NIRvascan [N]		Labspec 4i [N]	NIRvascan [N]
1 d (<10 d)	100 [40]	100 [62]	Unfed (0)	96.3 [134]	96.8 [158]
10 d (≥10 d)	81.3 [80]	69.5 [82]	Fed Once *	97.4 [77]	98.2 [111]
17 d (≥10 d)	100 [90]	100 [142]	Fed Twice *	86 [57]	97.8 [47]
Average	92.9 [210]	91.3 [286]	Average	94.4 [268]	97.4 [316]

d indicates age in days. [N] represents the total number of samples used. * Blood feeding history was grouped into fed or unfed regardless of the number of blood meals they received.

**Table 4:** Age prediction accuracy for Labspec 4i and NIRvascan for mosquitoes that were used to validate the models.

	Labspec	NIRvascan
	< or ≥10 Days Age Group	< or ≥10 Days Age Group
1 d	96.3 [81]	100 [58]
10 d	87.3 [118]	83.6 [116]
17 d	97.6 [167]	91.8 [116]
Average	94.0 [366]	90.0 [290]

**Table 5:** Prediction of blood feeding history using the Labspec 4i and NIRvascan spectrometers for mosquitoes within the test set.

Fed Condition	Predictive Accuracy [N]	
	Labspec 4i	NIRvascan
Unfed (0)	77.9 [199]	72.4 [174]
Fed Once (1) *	88.5 [61]	70.5 [68]
Fed Twice (2) *	95.8 [48]	68.9 [58]
Total Average	82.8 [308]	71.3 [300]

* Blood-feeding status was classified simply as unfed or blood-fed, regardless of the number of times a mosquito fed. [N] is the total number of samples.

**Table 6:** The effect of blood feeding on age prediction by Labspec 4i and NIRvascan spectrometers.

Blood Feeding Status	Age	Labspec 4i		NIRvascan	
		Mean Predicted Age	*p*-Value	Mean Predicted Age	*p*-Value
Unfed	10	14.3	0.001	14.5	0.049
Fed	10	12.3		13.2	
Unfed	17	15.2	0.005	14.5	0.678
Fed	17	14.1		14.8	

**Table 7:** Comparative analysis of the Labspec 4i and NIRvascan spectrometers in terms of time, cost, and operation.

Feature	NIRvascan	Labspec 4i
General configuration	** 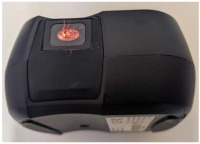 **	** 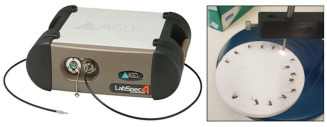 **
Size	82.2 × 66 × 45 mm, highly portable, lightweight (136 g)	127 × 356 × 292 mm, portable but larger than NIRvascan (5600 g)
Spectral range	900–1700 nm	350–2500 nm
Resolution	10 nm	- 3 @ 700 nm (Visible) - 10 @ 1400 nm (SWIR1) - 10 @ 2100 nm (SWIR2)
Current applications	- Agricultural monitoring - Food quality inspection - Pharmaceutical analysis - Recycling and material identification	- Mineral identification - Environmental analysis - Biological and agricultural research - Mosquito analysis
Average sampling time	30–45 s/sample	5–10 s/per sample
Average training time	10 min	30 min
Cost	USD 2695	USD 60,000
Advantages	- Easy to use - Portable - Ideal for in-field, rapid analysis - More affordable - Can be operated with a smartphone	- Broad spectral range - High signal-to-noise ratio - Versatile analysis modes - Sample scanning and prediction can be automated
Limitations	- Limited spectral range - Low signal-to-noise ratio - Scanning and prediction cannot be automated	- Less portable - Costly - Requires a laptop computer to operate

## Data Availability

The data presented in this study are only available on request from the corresponding author due to privacy reasons.
